# *Phytophthora infestans *small phospholipase D‐like proteins elicit plant cell death and promote virulence

**DOI:** 10.1111/mpp.12746

**Published:** 2018-10-16

**Authors:** Harold J. G. Meijer, Charikleia Schoina, Shutong Wang, Klaas Bouwmeester, Chenlei Hua, Francine Govers

**Affiliations:** ^1^ Laboratory of Phytopathology Wageningen University and Research PO Box 16 Wageningen 6700AA the Netherlands; ^2^ Wageningen Plant Research Wageningen University and Research PO Box 16 Wageningen 6700AA the Netherlands; ^3^ College of Plant Protection Agricultural University of Hebei Baoding 071001 China; ^4^ Present address: Center of Plant Molecular Biology (ZMBP) Eberhard‐Karls‐University Tübingen Tübingen D‐72076 Germany

**Keywords:** calcium, late blight disease, oomycete, phospholipases, phospholipids, signal peptide

## Abstract

The successful invasion of host tissue by (hemi‐)biotrophic plant pathogens is dependent on modifications of the host plasma membrane to facilitate the two‐way transfer of proteins and other compounds. Haustorium formation and the establishment of extrahaustorial membranes are probably dependent on a variety of enzymes that modify membranes in a coordinated fashion. Phospholipases, enzymes that hydrolyse phospholipids, have been implicated as virulence factors in several pathogens. The oomycete *Phytophthora infestans *is a hemibiotrophic pathogen that causes potato late blight. It possesses different classes of phospholipase D (PLD) proteins, including small PLD‐like proteins with and without signal peptide (sPLD‐likes and PLD‐likes, respectively). Here, we studied the role of sPLD‐like‐1, sPLD‐like‐12 and PLD‐like‐1 in the infection process. They are expressed in expanding lesions on potato leaves and during *in vitro* growth, with the highest transcript levels in germinating cysts. When expressed *in planta* in the presence of the silencing suppressor P19, all three elicited a local cell death response that was visible at the microscopic level as autofluorescence and strongly boosted in the presence of calcium. Moreover, inoculation of leaves expressing the small PLD‐like genes resulted in increased lesion growth and greater numbers of sporangia, but this was abolished when mutated PLD‐like genes were expressed with non‐functional PLD catalytic motifs. These results show that the three small PLD‐likes are catalytically active and suggest that their enzymatic activity is required for the promotion of virulence, possibly by executing membrane modifications to support the growth of *P. infestans *in the host.

## Introduction

Successful plant colonization by pathogens is the result of a complex interplay between the two partners and depends on the strict regulation of a variety of genes or the modification of cellular components, such as enzymes, structural proteins or lipids. For (hemi‐)biotrophic pathogens that form haustoria for the uptake of nutrients from the host and the delivery of effectors to the host, modifications of the host plasma membrane and the establishment of extrahaustorial membranes are crucial (Whisson *et al*., 2016). These pathogens presumably exploit certain enzymes to modify membranes in a coordinated fashion and, as such, these enzymes play a role as virulence factors. An example is phospholipase D (PLD), an enzyme that hydrolyses structural phospholipids resulting in the production of phosphatidic acid (PA), a second messenger that acts as a mediator in many cellular processes. Phospholipases have been implicated as virulence factors in several pathogens (Ghannoum, 2000; Köhler *et al*., 2006).

The oomycete *Phytophthora infestans *is the causal agent of late blight on potato and tomato, and is also known as the Irish potato famine pathogen. It is a hemibiotrophic pathogen that penetrates leaves after breaching the plant cuticle, and then expands as intracellular mycelium whilst forming haustoria in mesophyll cells. *P. infestans* possesses a large repertoire of PLDs divided over six subfamilies, including two families of small PLD‐likes with signal peptide (referred to as secreted PLDs or sPLD‐likes) (Meijer *et al*., 2011). Their function is still unknown, but the finding that PLD activity can be recovered from liquid medium harbouring *P. infestans *mycelium suggests that these small PLDs are secreted and potentially capable of modifying host tissue (Meijer *et al*., 2011, 2014).

Phospholipids, the substrates of PLDs, are ubiquitous components of membranes and are functionally involved in a disproportionately large number of cellular processes. In addition to regulating membrane‐bound enzymatic activities, they act as anchors to recruit and translocate proteins containing a phospholipid‐binding module and play essential roles in membrane trafficking and defence signalling (Behnia and Munro, 2005, Lemmon, 2003, Meijer and Munnik, 2003, Testerink and Munnik, 2011). PA, the product of PLD activity, is an important intermediate in lipid biosynthesis and a signalling molecule involved in vesicle formation and transport, cytoskeleton organization, protein transport, signal transduction and mitosis, etc. (Testerink and Munnik, 2005, 2011; Zhang *et al*., 2012). Direct application of PA to plant leaves results in pathogenesis‐related gene expression and cell death (Andersson *et al*., 2006; Park *et al*., 2004; Testerink and Munnik, 2011).

Eukaryotic PLDs usually have two catalytic conserved motifs, each with the core sequence ‘HxKxxxxD’, referred to as the HKD1 and HKD2 motifs (IPR001736). These catalytic sites can be surrounded by accessory regulatory domains, as is the case in the two major PLD families, PXPH‐PLDs and C2‐PLDs, which have a phospholipid‐binding domain (PXPH or C2) at the N‐terminus (Selvy *et al*., 2011). In the φ‐type PLD subfamily, canonical regulatory domains are lacking but, instead, many members have signal peptides (Beligni *et al*., 2015; Liu *et al*., 2010; Tang *et al*., 2016). *Phytophthora *spp. have six PLD subfamilies (Meijer and Govers, 2006; Meijer *et al*., 2011) that are well conserved amongst oomycetes (Baxter *et al*., 2010; Jiang *et al*., 2013; Kemen *et al*., 2011; Levesque *et al*., 2010; Sharma *et al*., 2015). They are classified as PXPH‐PLD, PXTM‐PLD, TM‐PLD, sPLD‐like‐type‐A (φ‐type), sPLD‐like‐type‐B and PLD‐like. Oomycetes have no C2‐PLDs, which, so far, have only been found in plants, but, instead, have two novel oomycete‐specific PLDs, i.e. PXTM‐PLD and TM‐PLD, with a transmembrane region as accessory domain (Meijer and Govers, 2006; Meijer *et al*., 2005). In *P. infestans*, the first four of the six subfamilies contain only a single gene, whereas the last two are multigene families. The three PLD‐likes are homologous to the nine sPLD‐like‐type‐Bs, but lack a signal peptide (Meijer *et al*., 2011). Of the six classes, three have two perfect catalytic HKD motifs. In TM‐PLD, HKD2 is changed in HKN, and, in the PLD‐likes and sPLD‐like‐Bs, HKD1 is modified in either HKL, HKT, HKA or HKR (Meijer *et al*., 2011). Such imperfect HKD motifs are also found in distinctly related PLD‐likes from actinomycetes, in particular *Janibacter* spp. and *Kineococcus radiotolerans*. Despite the aberrant HKD1 motif, the PLD‐likes and sPLD‐like‐Bs share other typical PLD motifs with PXPH‐PLDs and C2‐PLDs (Meijer *et al*., 2011), pointing to similarities in catalytic activity.

In several bacterial pathogens, PLDs are implicated as major virulence determinants. For example, in *Corynebacterium pseudotuberculosis*, PLDs are critical for the dissemination of this pathogen from the infection site to the lymph nodes (McKean *et al*., 2007). Moreover, disruption of a PLD gene in *Acinetobacter baumannii* and *Helicobacter pylori* strongly diminishes pathogenicity (Jacobs *et al*., 2010, Sitaraman *et al.*, 2012). Also, in *Klebsiella pneumoniae*, a PLD acts as a virulence factor, presumably by controlling the bacterial membrane lipid composition (Lery *et al*., 2014). In fungi, PLD involvement in pathogenicity has been demonstrated in *Candida albicans*, *Purpureocillium lilacinum *and *Coccidioides posadasii* (Dolan *et al*., 2004; Hube *et al*., 2001; Lajoie and Cordes, 2015; Yang *et al*., 2015).

Previously, we have reported the recovery of significant levels of PLD activity in liquid medium supporting the *in vitro* growth of *P. infestans. *The activity is dependent on the nutrient content of the medium, suggesting that the pathogen senses the environment and adapts accordingly (Meijer *et al*., 2011, 2014). Likewise, it can be hypothesized that the pathogen senses its host and is triggered to secrete PLD proteins to execute host membrane modification. In this study, we focus on potentially secreted PLDs of the subfamilies sPLD‐like‐A and sPLD‐like‐B, and include a PLD‐like because of its close resemblance to sPLD‐likes of type B (Fig. S1, see Supporting Information). As all PLD‐likes and sPLD‐likes (united as (s)PLD‐likes) are relatively small (maximum of 68 kDa), they are also collectively referred to as small PLD‐likes. Here, we report that small PLD‐like genes are expressed in germinating cysts and during plant infection. When testing the effect of small PLD‐likes on *Nicotiana benthamiana*, we found that, on the one hand, they elicit cell death in a calcium‐dependent manner and, on the other, promote the virulence of *P. infestans. *Mutations in the catalytic HKD motifs strongly reduce the cell death responses and abolish virulence promotion, demonstrating that the enzymatic activity of the PLDs is the major determinant. These data strengthen the idea that small PLD‐likes act as pathogenicity factors in *P. infestans*.

## Results

### Differential expression of small PLD‐likes during development and leaf colonization

**Figure 1:**
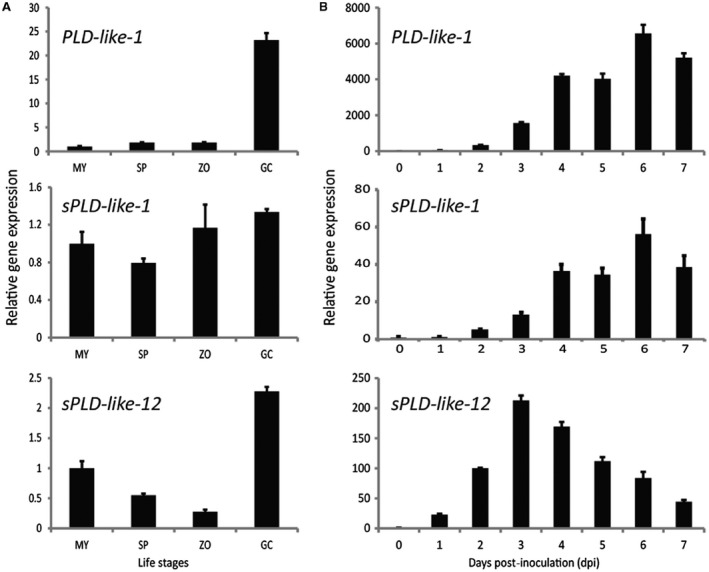
Expression profiles of small phospholipase D (PLD)‐like genes of *Phytophthora infestans* during asexual development (A) and growth *in planta *after inoculation on potato leaves (B). Gene expression was analysed by quantitative reverse transcription‐polymerase chain reaction (qRT‐PCR) relative to the expression in mycelium (set at 1), with *P. infestans ActA* as endogenous control. Error bars represent the standard error of two biological repeats. GC, germinating cysts; MY, mycelium; SP, sporangia; ZO, zoospores.

We selected PLD‐like‐1 (PITG_00921), sPLD‐like‐1 (PITG_18185) and sPLD‐like‐12 (PITG_12806), representing each small PLD‐like class in *P. infestans* (Haas *et al*., 2009; Meijer *et al*., 2011) (Fig. S1). The expression of *PLD‐like‐1*, *sPLD‐like‐1* and *sPLD‐like‐12* was determined by quantitative reverse transcription‐polymerase chain reaction (qRT‐PCR) in four developmental stages and during *in planta *growth. For *sPLD‐like‐1*, expression levels were similar in mycelium, sporangia, zoospores and germinating cysts with less than two‐fold variation between the four stages (Fig. 1A). However, the expression of *PLD‐like‐1* and *sPLD‐like‐12* was highest in germinating cysts with a 23.3‐ and 2.3‐fold increase when compared with the mycelial stage, respectively (Fig. 1A). On inoculation of potato leaves with *P. infestans *zoospores and during subsequent disease development, the expression of *PLD‐like‐1* and *sPLD‐like‐1* started to increase at 2 days post‐inoculation (dpi), peaked at 6 dpi and thereafter declined (Fig. 1B). The expression of *sPLD‐like‐12* started to increase earlier, at 1 dpi, and also peaked earlier, at 3 dpi, before declining (Fig. 1B).

These results show that each of the three small PLD‐likes has its own distinct expression pattern, with *PLD‐like‐1* showing the highest relative increase in expression in germinating cysts and even higher in expanded lesions. In contrast, *sPLD‐like‐12* shows a more modest increase in germinated cysts and peaks relatively high, shortly after inoculation, in an early stage of infection. Of the three small PLD‐likes, *sPLD‐like‐1* shows the least strong fluctuation in expression, but, nevertheless, similar to *PLD‐like‐1*, its expression peaks at 6 dpi and gradually increases during lesion expansion.

### Expression of small PLD‐likes *in planta* results in local cell death

**Figure 2:**
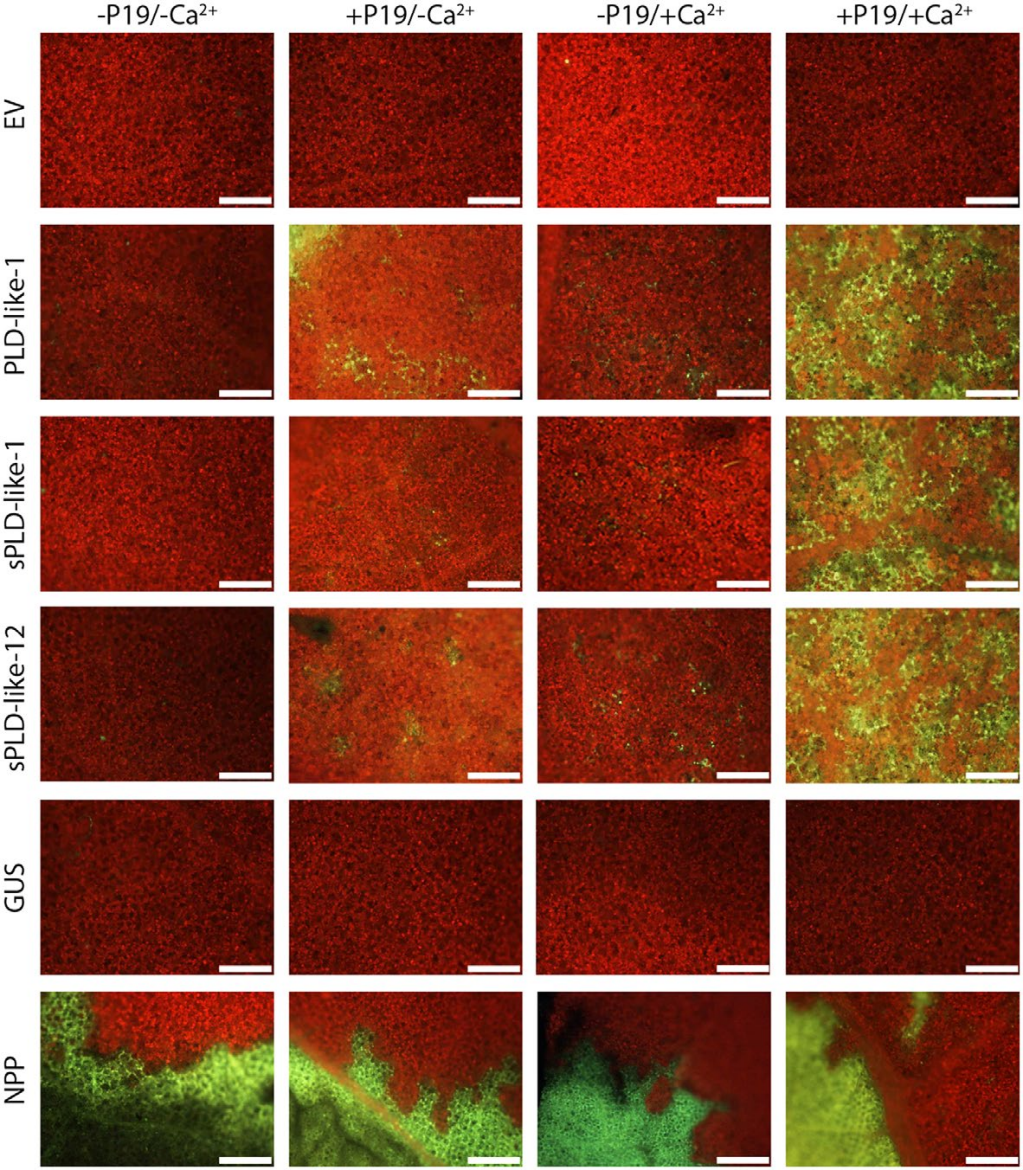
Small phospholipase D (PLD)‐likes induce cell death in *Nicotiana benthamiana.* Epifluorescence photographs of leaves at 7 days after agroinfiltration with *Agrobacterium tumefaciens* Agl1 carrying small PLD‐like constructs (PLD‐like‐1, sPLD‐like‐1 and sPLD‐like‐12) and control constructs [empty vector (EV), β‐glucuronidase (GUS) and NPP]. Autofluorescence of dead cells is depicted in yellow–green and that of living cells in red. Cell death is enhanced by co‐expression of the silencing suppressor P19 (+P19) and in the presence of calcium (+Ca^2+^; infiltration of 2 mm CaCl_2_ at 24 h after agroinfiltration). Experiments were repeated three times (*n* = 24). Scale bars represent 500 μm.

The three small PLD‐like genes from *P. infestans *were expressed in *N. benthamiana* leaves by agroinfiltration with *Agrobacterium tumefaciens* strain Agl1 containing the binary vector pGRAB with inserts encoding PLD‐like‐1, sPLD‐like‐1 or sPLD‐like‐12. When infiltrated, the leaves showed no obvious response when compared with the negative controls, empty vector (EV) or β‐glucuronidase (GUS) (Fig. 2). In contrast, the positive control, pGRAB carrying the *PiNPP1*(necrosis and ethylene‐inducing peptide 1‐like protein) gene, which encodes a general necrosis‐inducing elicitor, caused tissue collapse, as reported previously (Kanneganti *et al*., 2006; Wang *et al*., 2015). As this lack of response could be a result of low expression levels of the (s)PLD‐like genes, we exploited P19, a viral‐encoded suppressor of gene silencing, that prevents the onset of post‐transcriptional gene silencing and allows high levels of transient expression in agroinfiltrated leaves (Voinnet *et al*., 2003). Co‐infiltration of P19 with PLD‐like‐1, sPLD‐like‐1 or sPLD‐like‐12 resulted in cell death within 6 days. Figure 2 shows the response visualized by microscopy and observed as autofluorescence at 7 days after infiltration. Although this cell death response was relatively weak when compared with the positive control PiNPP1, no response was observed when the negative controls were co‐infiltrated with P19. In addition, the number of spots reminiscent of cell death fluctuated for each individual leaf, but was consistently distinguishable from that of the empty vector control (*n* > 20).

### PLD‐induced cell death is more pronounced in the presence of calcium

Several PLDs, derived from a variety of organisms, require calcium for full activity. The optimum calcium concentration ranges from micromolar to millimolar levels depending on the type of PLD (Li *et al*., 2009; Meijer and Munnik, 2003; Testerink and Munnik, 2011). To test whether the three *P. infestans *small PLD‐likes are calcium dependent, we re‐infiltrated the leaves with 2 mm CaCl_2_ at 24 h after agroinfiltration with Agl1 strains carrying the PLD constructs. In a pilot experiment, in which we tested a range of CaCl_2_ concentrations, we determined 2 mm as the optimal concentration. With calcium as stimulating factor, local cell death was visible in leaves expressing the small PLD‐likes, but not in leaves infiltrated with the negative control EV. The cell death response was similar to that observed on co‐infiltration with P19 (Fig. 2). However, combining the calcium treatment with the P19 silencing suppressor significantly boosted the cell death response (Fig. 2). Despite this boost, tissue collapse was still less prominent than in leaves infiltrated with NPP1.

**Figure 3:**
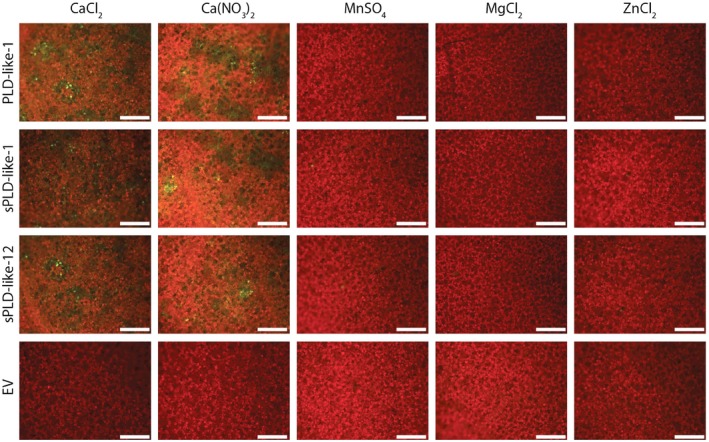
Cell death induced by small phospholipase D (PLD)‐likes is enhanced in the presence of calcium. Epifluorescence photographs of *Nicotiana benthamiana* leaves at 7 days after agroinfiltration with *Agrobacterium tumefaciens* Agl1 carrying small PLD‐like constructs (PLD‐like‐1, sPLD‐like‐1 and sPLD‐like‐12) and the control construct EV (empty vector). One day after agroinfiltration, the leaves were infiltrated with 2 mm solutions containing the different compounds as indicated in the figure. Dead cells are depicted in green and living cells in red. Experiments were repeated three times (*n* = 24). Scale bars represent 500 μm.

To investigate whether calcium is the only ion capable of stimulating cell death, we also tested the activity of magnesium, manganese and zinc. At 24 h after co‐agroinfiltration (PLD constructs and P19), leaves were re‐infiltrated with 2 mm CaCl_2_, Ca(NO_3_)_2_, MnSO_4_, MgCl_2_ or ZnCl_2_. Microscopic examination revealed that calcium‐based salts provoked cell death, whereas non‐calcium‐based salts were ineffective (Fig. 3). Apparently, the capacity of small PLD‐likes to induce cell death is enhanced specifically by calcium. To further investigate the importance of calcium, the infiltrations were repeated in the presence of lanthanum chloride. La^3+^ is a potent Ca^2+^ channel blocker and acts as a calcium antagonist. In the presence of 100 µm LaCl_3_, the cell death boosted by 2 mm CaCl_2_ was strongly reduced (Fig. S2, see Supporting Information). In conclusion, the cell death‐inducing activity of small PLD‐likes is highly dependent on the presence of calcium.

### The signal peptide is essential for the cell death‐inducing activity of sPLD‐likes

**Figure 4:**
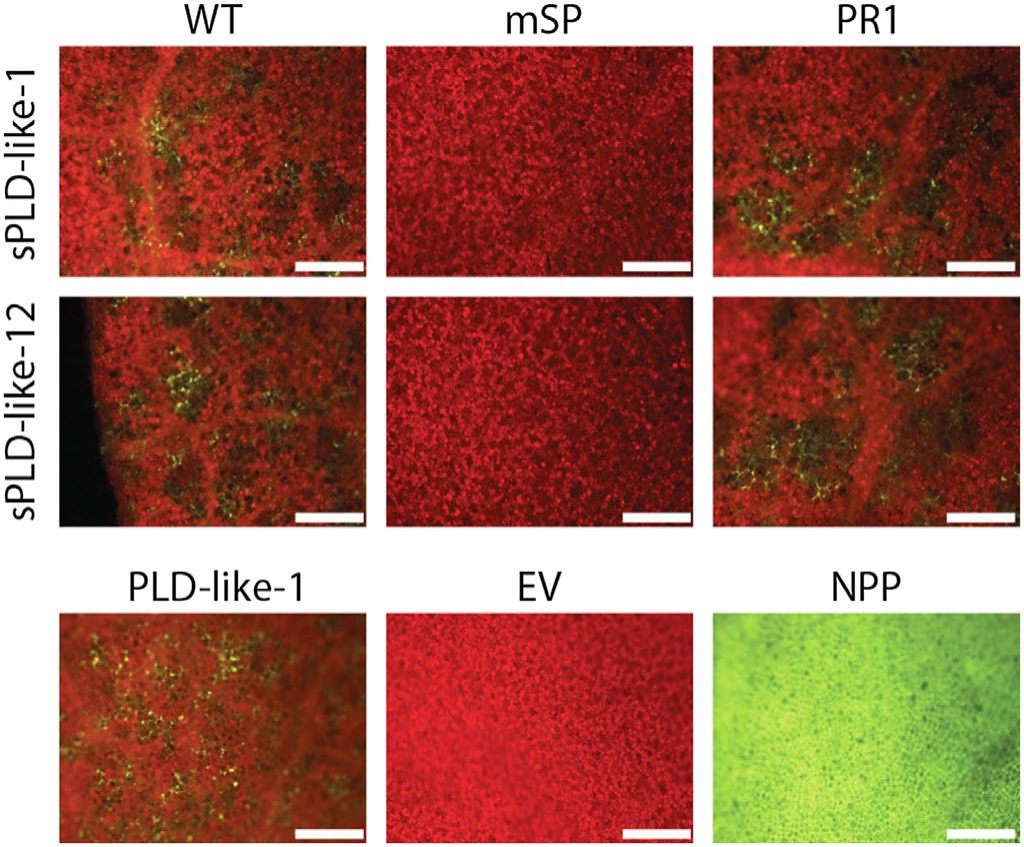
The secretion of sPLD‐like‐1 and sPLD‐like‐12 is required for cell death induction. Epifluorescence photographs of *Nicotiana benthamiana* leaves at 7 days after agroinfiltration with *Agrobacterium tumefaciens* Agl1 carrying wild‐type sPLD‐like‐1 and sPLD‐like‐12 constructs (WT), constructs lacking the signal peptide (mSP) or with the signal peptide replaced by the signal peptide from tobacco PR1 (PR1), and control constructs [phospholipase D (PLD)‐like‐1, empty vector (EV) and NPP]. P19 was co‐expressed and 2 mm CaCl_2_ was infiltrated at 24 h after agroinfiltration. Dead cells are depicted in green and living cells in red. Experiments were repeated three times (*n* = 24). Scale bars represent 500 μm.

Based on signal peptide prediction programs, a highly supported signal peptide is predicted for sPLD‐like‐12, whereas, for sPLD‐like‐1, the prediction is relatively weak (Meijer *et al*., 2011). To test whether the signal peptides of the two sPLD‐likes are essential for cell death‐inducing activity, truncated sPLD‐like constructs were generated in which we deleted the signal peptide (mSP). Agroinfiltration of these mSP constructs failed to induce cell death, even in the presence of P19 and calcium (Fig. 4). To obtain further evidence that secretion is essential for activity, we infiltrated sPLD‐like constructs in which the authentic signal peptide was replaced by the signal peptide of the extracellular pathogenesis‐related protein PR1 (Pfitzner and Goodman, 1987). This resulted in local cell death responses comparable with those observed for wild‐type (s)PLD‐likes (Fig. 4). These results suggest that the weakly predicted signal peptide in sPLD‐like‐1 is indeed functional as a signal peptide. With respect to sPLD‐like‐12, the strong effect of the signal peptide was less anticipated. This type B sPLD‐like shares over 50% sequence identity with PLD‐like‐1 (Meijer *et al*., 2011) and, as shown above, the latter induces cell death whilst lacking a signal peptide.

### Induction of cell death is dependent on intact catalytic motifs

**Figure 5:**
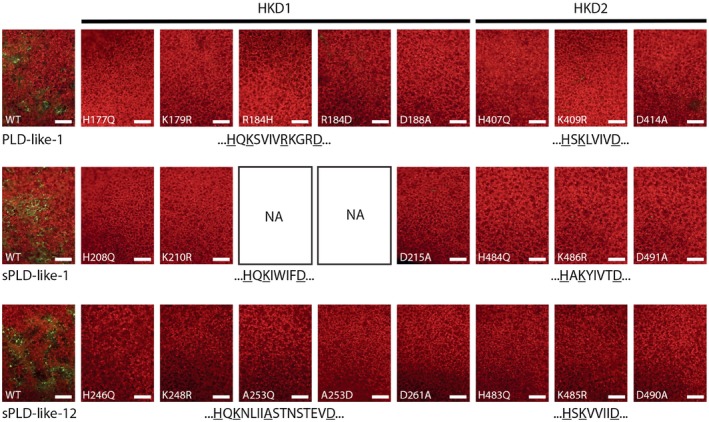
Mutations in the HKD motifs of the small phospholipase D (PLD)‐likes abolish cell death‐inducing activity. Epifluorescence photographs of *Nicotiana benthamiana* leaves at 7 days after agroinfiltration with *Agrobacterium tumefaciens* Agl1 carrying constructs with mutated versions of the small PLD‐likes. P19 was co‐expressed and 2 mm CaCl_2_ was infiltrated at 24 h after agroinfiltration. Dead cells are depicted in yellow–green and living cells in red. Experiments were repeated three times (*n* = 24). Scale bars represent 500 μm. NA, not applicable. WT, wild‐type.

Amino acid substitutions in the catalytic domain of PLDs have been proven to be destructive for their catalytic activity (Sung *et al*., 1997). To test whether the cell death‐inducing activity of each of the small PLD‐likes depends on the HKD motif, all key amino acids were independently mutated (Table 1). In the case of sPLD‐like‐1, both HKD motifs are according to the core sequence. PLD‐like‐1 and sPLD‐like‐12 lack the aspartate residue (D) of HKD1. Therefore, we substituted the amino acids according to the positions in the core HKD motif plus one additional amino acid. In PLD‐like‐1, an adjacent aspartate (D188) residue was selected for mutation because it was hypothesized to replace the arginine at position 184 (R184). Similarly, in sPLD‐like‐12, the aspartate residue at position 261 (D261) was selected for mutation as it could be the substitute for alanine located at position 253 (A253). Agroinfiltration of the substitution constructs in the absence or presence of P19/Ca^2+^ did not reveal any activity. Cell death was undetectable, or at least extremely limited and comparable with the negative control. In contrast, the wild‐type small PLD‐likes, used as positive controls, induced local cell death as expected (Fig. 5; Table 1).

**Table 1:**
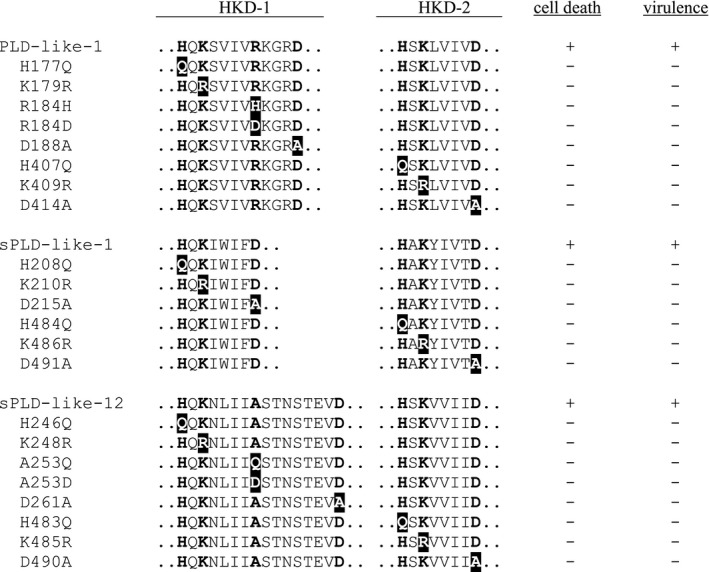
Amino acid substitutions in the HKD1 and HKD2 catalytic motifs of the small phospholipase D (PLD)‐likes and the effect of these substitutions on cell death induction and virulence.

**Figure 6:**
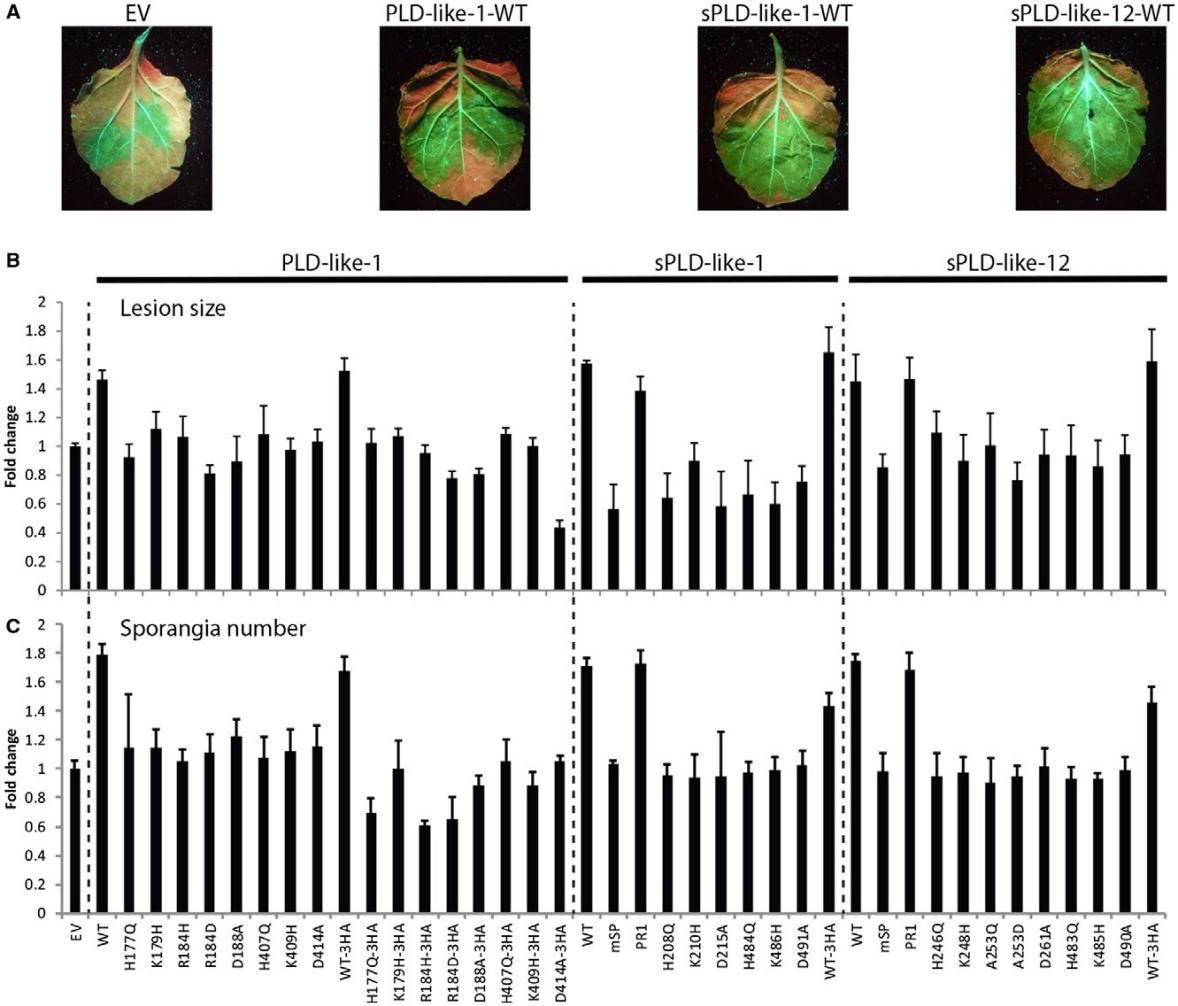
Small phospholipase D (PLD)‐likes promote *Phytophthora infestans *growth and sporulation. (A) Representative UV photographs of agroinfiltrated *Nicotiana benthamiana* leaves infected with *Phytophthora infestans* strain 14‐3‐GFP at 7 days post‐inoculation. WT, wild‐type. Lesion size (B) and sporulation (C) of *P. infestans *on leaves infiltrated with various constructs of small PLD‐likes, calculated as the fold change in lesion size or amount of sporangia, respectively, compared with that on leaves infiltrated with the control construct (EV). P19 was co‐expressed and 2 mm CaCl_2_ was infiltrated at 24 h after agroinfiltration. Experiments were repeated three times. Error bars represent standard deviation (*n* = 24).

The lack of cell death‐inducing activity for all modified small PLD‐likes urged us to reconsider whether the amino acid substitutions could have an effect on the stability of the proteins. Based on the nature of the substitutions, it is unlikely that they affect the overall protein structure (Sung *et al*., 1997). To detect the proteins, we added a 3HA (HA, haemagglutinin) tag to the C‐terminus of the PLDs and checked whether the activity of the three wild‐type PLDs was affected by the presence of the tag. Agroinfiltration confirmed that all three 3HA‐tagged wild‐type small PLD‐likes induced cell death at similar levels to the non‐tagged versions in the presence of Ca^2+^ (Fig. 6A). Proteins isolated from total leaf extracts, agroinfiltrated with the constructs but in the absence of Ca^2+ ^to avoid cell death, were then analysed for the presence of HA‐tagged proteins by Western blot analyses. PLD‐like‐1‐3HA was detected, but sPLD‐like1‐3HA and sPLD‐like‐12‐3HA remained undetected (Fig. S3C, see Supporting Information). The latter suggests that the HA‐tagged proteins are present at extremely low levels, unstable during the extraction procedure or the tag is cleaved during protein processing. We failed to isolate tagged (s)PLD‐likes from the apoplastic fluid of agroinfiltrated leaves.

Subsequently, substitution constructs of PLD‐like‐1 fused to a 3HA tag were tested for their cell death‐inducing capacity. None of the substitution constructs induced cell death on agroinfiltration in the presence of P19 and calcium, whereas, in five of nine, the corresponding protein was detected in extracts of infiltrated leaves (Fig. S3B,D). As the HKD versions of sPLD‐like‐1 and sPLD‐like‐12 harbour similar amino acid substitutions, we anticipate that most of the mutated PLDs are produced, albeit at extremely low levels, similar to that found for the wild‐type PLD‐likes. Nevertheless, there was no cell death, and this is a strong indication that the catalytic activity of the small PLD‐likes is important for inducing cell death.

### *In planta *expression of small PLD‐likes promotes *P. infestans *growth and sporulation

To investigate whether or not small PLD‐likes play a role in the virulence of *P. infestans*, we tested the effect of heterologous expression of small PLD‐likes in *N. benthamiana* on lesion growth. Leaves were agroinfiltrated with the various PLD constructs described above. After 2 days, leaves were detached and inoculated with *P. infestans *strain 14‐3‐GFP (Du *et al*., 2015). Lesions on leaves agroinfiltrated with the wild‐type small PLD‐likes were significantly larger than those agroinfiltrated with EV [one‐way analysis of variance (ANOVA), *P* < 0.05, Fig. 6A,B]. This was accompanied by enhanced sporulation as more sporangia were produced compared with the control (Fig. 6C). Deletion of the signal peptide of sPLD‐like‐1 and sPLD‐like‐12 resulted in the loss of both lesion growth promotion and enhanced sporulation, whereas replacement by the PR1 signal peptide partially restored these features (Fig. 6B,C). In addition, the HKD substitution constructs failed to promote lesion growth and sporangia production; the effects were in line with those of the controls (EV and non‐infiltrated leaves; Fig. 6B,C). Similarly, all HA‐tagged small PLD‐likes, both wild‐type and mutants, behaved as their non‐tagged counterparts (Fig. 6B,C). Strikingly, the growth promotion and enhanced sporulation were also observed when the agroinfiltrations were performed in the absence of P19 and/or calcium (Fig. S4, see Supporting Information), conditions that boosted (s)PLD‐likes with respect to their cell death‐inducing activity. It could well be that *P. infestans *modulates the leaf environment to create optimal conditions for its own secreted enzymes*, *including its endogenous sPLD‐likes. The (s)PLD‐likes expressed in the plant cell via agroinfiltration may also benefit from this modulation and hence no longer need the extra boost of calcium and/or P19.

Altogether, these results suggest that sPLD‐likes enter the secretory pathway in order to execute their growth‐promoting function and that, in all three small PLD‐likes, a functional HKD motif is required to promote growth and sporulation.

## Discussion

In this study, we tested the hypothesis that small PLD‐likes produced by oomycetes act as virulence factors. Incentives for this study were the findings that PLDs and secreted PLDs have been implicated as major virulence determinants in bacteria and fungi (Dolan *et al*., 2004; Hube *et al*., 2001; Jacobs *et al*., 2010; Lajoie and Cordes, 2015; Lery *et al*., 2014; McKean *et al*., 2007; Sitaraman *et al*., 2012; Yang *et al*., 2015), and the fact that oomycetes have an extended repertoire of PLDs, including subfamilies with novel oomycete‐specific PLDs and potentially secreted PLDs (Meijer and Govers, 2006; Meijer *et al*., 2011).

For our experiments, we selected three candidates from the 13 small PLD‐likes that were identified in *P. infestans, *each representing a distinct subfamily. sPLD‐like‐1 is the only sPLD‐like type A in *P. infestans *and is quite divergent from all other PLDs. sPLD‐like‐A genes are widespread in the tree of life and probably share an ancient ancestor. The closest relatives of sPLD‐like‐1 are found in plants (Meijer *et al*., 2011). sPLD‐like‐12 is one of the nine members in the sPLD‐like‐B subfamily in *P. infestans*, whereas the closely related PLD‐like‐l belongs to a PLD‐like subfamily with three members. Unlike sPLD‐like‐Bs, PLD‐likes lack a signal peptide (Meijer *et al*., 2011)*. *All PLD subfamilies are well conserved amongst oomycetes, always with multiple copies of sPLD‐like‐Bs and PLD‐likes, albeit with some variations in numbers (Baxter *et al*., 2010; Jiang *et al*., 2013; Kemen *et al*., 2011; Levesque *et al*., 2010; Sharma *et al*., 2015). The clustering of sPLD‐like‐Bs and PLD‐likes and the great distance to other PLDs are reminiscent of expanded oomycete‐specific protein families whose members have putative functions in virulence or pathogenicity.

A prerequisite for acting as a virulence factor is the expression of the encoding gene during interaction of the pathogen with its host. The expression of all three small PLD‐like genes was found to be up‐regulated during *in planta* growth in potato leaves. *PLD‐like‐1*, one of the highest transcribed genes in *P. infestans* (Meijer *et al*., 2011), peaks at 6 dpi when the interaction has already reached the necrotrophic stage. In addition, *sPLD‐like‐1* peaks at 6 dpi, but, in contrast, *sPLD‐like‐12* is induced shortly after inoculation with a peak at 3 dpi, presumably functioning in the early biotrophic stage of the interaction. Most sPLD‐like genes, including *sPLD‐like‐1* and *sPLD‐like‐12*, also showed increased expression during tuber infections (Ah‐Fong *et al*., 2017). Moreover, during infection of tomato leaves, expression of *PLD‐like‐1* and *sPLD‐like‐1 *(but not of *sPLD‐like‐12*) was monitored and also found to be up‐regulated (Zuluaga *et al*., 2016). In both the biotrophic and necrotrophic stages, *P. infestans *sporulates abundantly and, as such, the sporulation‐promoting activity (Fig. 6) of the three small PLD‐likes might be beneficial for the pathogen throughout the interaction.

PA, the product of hydrolysis of phospholipids catalysed by PLD, is a multifunctional second messenger that is frequently generated during stress conditions. To boost the production of PA during infection, pathogens can either hijack PLDs from the host, a strategy used by the *Red clover necrotic mosaic virus* in *N. benthamiana* (Hyodo *et al*., 2015), or can produce their own PLDs, which then exploit plant phospholipids as substrates. The finding that strong PLD activity was found in the extracellular medium of *P. infestans* (Meijer *et al*., 2011, 2014) suggests that this pathogen secretes PLDs, instrumental in the colonization of the host. To test how the plant responds to pathogen‐derived PLDs, we expressed the small PLD‐like genes *in planta* and monitored the responses. The responses, observed as localized cell death, were enhanced in the presence of calcium, a well‐known stimulator of PLD activity (Li *et al*., 2009; Meijer and Munnik, 2003; Testerink and Munnik, 2011). The replacement of calcium with other ions or the addition of the Ca^2+^ channel blocker La^3+^ resulted in a strongly reduced cell death, showing indirectly that PLD activity is the trigger for cell death. This was further confirmed by the finding that the *in planta* expression of small PLD‐like genes with point mutations affecting the HKD motifs failed to induce cell death. For PLD‐like‐1, we demonstrated by Western blot analyses that this loss of activity was not a result of the loss of overall stability of the mutated versions of the protein. Five of nine HKD mutated versions of PLD‐like‐1 were found to be stably present in infiltrated leaves without causing cell death. For sPLD‐like‐1 and sPLD‐like‐12, we could not demonstrate this because the 3HA‐tagged versions of these two small PLD‐likes were not detectable on Western blots, either in total extracts from agroinfiltrated leaves or in apoplastic fluid. Yet, as most of the mutated PLD‐like‐1 versions are stable, we assume that the other mutated small PLD‐likes are also stable; in all cases, similar amino acids are changed in motifs that are highly conserved. Moreover, it is well established that such targeted modifications of catalytic sites of PLDs significantly lower phospholipid turnover or simply render the enzyme inactive (Rudolph *et al*., 1999; Sung *et al*., 1997). Altogether, we conclude that the induction of cell death by the small PLD‐likes fully depends on catalytic activity.

The observation that the three small PLD‐likes elicited similar responses irrespective of the signal peptide raised the question of where the PLD activity resides. Based on the finding that the removal of the signal peptide abolished activity, it might be concluded that, in the agroinfiltrated leaves, sPLD‐like‐1 and sPLD‐like‐12 are secreted in the apoplast, the site at which they readily end up during natural infections when secreted by *P. infestans*. The conventional transport of secreted proteins is via the endoplasmic reticulum, a main Ca^2+^ storage organelle (Denecke, 2007). PLD‐like‐1 is leaderless, but could still be secreted via an unconventional secretion pathway. Many organisms have such pathways, and *P. infestans *does too, as several proteins lacking a signal peptide were identified in the secretome (Meijer *et al*., 2014). However, there are also arguments in favour of the hypothesis that the small PLD‐likes are functional inside plant cells. One argument is the stimulating influence of calcium on all three small PLD‐likes, including that lacking a signal peptide, combined with the inhibitory effect on cell death by La^3+^, a Ca^2+^ channel blocker. As Ca^2+^ channels are located in the plasma membrane or in intracellular membranes, La^3+^ is not expected to affect PLD activity in the apoplast. Another argument is the occurrence of cell death in one or just a few cells. In general, the cell death response was relatively weak and, in contrast with the full tissue collapse elicited, for example, by NPP1 (Kanneganti *et al*., 2006; Wang *et al*., 2015) or INF1 (Kamoun *et al*., 1997), cell death was limited to individual cells or small cell clusters spread over the infiltrated area. This suggests that minute quantities of PLDs are sufficient to induce cell death, thereby restricting the diffusion of proteins to adjacent cells, as would be expected when PLDs enter the apoplast. Another argument, although less strong, is that the only 3HA‐tagged small PLD‐like detectable in total leaf extracts by Western blotting was not detected in apoplastic fluid. If this is the case, the next question is: how do the PLDs enter the host? Many pathogens, including *Phytophthora *spp., have the ability to translocate effectors into the host cell, where they function as virulence factors, mainly by suppressing defence responses. Such effectors have a characteristic RXLR motif in the N‐terminal part that is essential for translocation. None of the PLDs, including those from the other subfamilies not studied here, contain such a motif.

To assess the role of the small PLD‐likes during host infection, we tested whether the presence of higher levels would be beneficial for *P. infestans *growth. This revealed that all three small PLD‐likes facilitate *P. infestans *growth, resulting in the development of larger lesions and more sporangia. In contrast, the presence of the HKD mutated versions of the PLDs had no effect, demonstrating that promotion of virulence by small PLD‐likes depends on intact catalytic domains. The presence of calcium and/or the silencing suppressor did not further increase the virulence‐promoting activity. Possibly, calcium was not limited because of the local increase in calcium levels that is frequently reported to occur during plant–pathogen interactions (Zhang *et al*., 2014).

The *in vivo *role of small PLD‐likes remains to be elucidated. The cell death‐inducing and growth‐promoting features might turn out to be extrapolations of their enzymatic activity. During infection, the plasma membranes of infected plant cells are modified at sites at which haustoria are formed and invade the host cell (Micali *et al*., 2011). These changes in structure and physiology are initiated by the pathogen to promote the exchange of proteins and nutrients without provoking immediate cell death. This study strongly supports our hypothesis that small PLD‐likes play a role in virulence. We anticipate that, under natural conditions, they are deployed by *P. infestans* to modulate plant membranes, thereby facilitating invasion and colonization. Their activity can result in localized PA production, e.g. on the extrahaustorial membrane or any membrane, or influence membrane curvature, and hence lead to recruitment and dynamic regulation of the activity of a broad spectrum of molecules (Putta *et al*., 2016). We assume that, as a group, the small PLD‐likes can synergistically enhance the pathogenic capacity of *P. infestans*.

## Experimental Procedures

### *Phytophthora infestans* strains and material

*Phytophthora infestans* strains 88069 and 14‐3‐GFP were maintained on rye sucrose medium at 18 ℃. Mycelium, sporangia, zoospores and germinated cysts for RNA isolation were obtained as described previously (Hua *et al*., 2013) and stored at −80º℃.

### Plant material and infection assays

Potato (cultivar Bintje, R0) and *N. benthamiana* plants were grown in a glasshouse under standardized conditions. Infected plant material for RNA isolation was obtained by inoculation of leaves detached from 6‐week‐old potato plants with 20 µL of a zoospore suspension (1 × 10^4^ zoospores/mL) of *P. infestans* strain 88069, followed by incubation in a climate chamber at 18 ℃ and high relative humidity. Leaves were harvested at seven consecutive days after inoculation, quickly frozen in liquid N_2_ and stored at −80 ℃.

### RNA extraction and qRT‐PCR

RNA from *P. infestans* material was extracted using TriZol (Invitrogen, Landsmeer, The Netherlands). RNA from infected and healthy potato leaves was isolated using a NucleoSpin RNA II RNA Extraction Kit (Macherey‐Nagel, Düren, Germany) following the procedures described by the manufacturer. To remove genomic DNA contamination, total RNA was treated with DNaseI, followed by a phenol–chloroform extraction. For qRT‐PCR, first‐strand cDNA was synthesized from 2 µg of total DNAse‐treated RNA using oligodT and a SuperScript First‐Strand Synthesis System (Invitrogen). PCR was performed with an ABI 7300 Real‐Time , Foster City, CA, USA) using SYBR‐GREEN Master Mix (Bio‐Rad, Hercules, CA, USA) (95 ℃ for 5 min; 40 cycles of 30 s at 95 ℃, 30 s at 60 ℃ and 20 s at 72 ℃; followed by 10 min at 72 ℃). Gene‐specific primers are listed in Table S1 (see Supporting Information). Each qRT‐PCR was performed in triplicate, with three biological repeats, and gene expression was normalized according to *P. infestans *actin A (*PiActA*) expression (Du *et al*., 2015; Hua *et al*., 2013).

### Plasmid construction

The primers used for cloning, the generation of mutations and tagging of PLD genes are listed in Table S1. Small PLD‐like genes were amplified from genomic DNA isolated from *P. infestans* strain 88069 (Haas *et al*., 2009; Meijer *et al*., 2011). Amplification was performed with proofreading *Pfu* polymerase (Promega, Leiden, The Netherlands). Fusion genes (PR1/HA‐tagged) and mutated versions were produced by overlap PCR. Fragments were purified from gel and cloned into pGEM‐T Easy vectors (Promega) that were transformed into *Escherichia coli* strain DH5. Inserts were sequenced using standard M13 primers (Macrogen Europe, Amsterdam, The Netherlands) and cloned into the binary vector pGRAB (Whisson *et al*., 2007), which was transformed into *A. tumefaciens* Agl1 strain.

### Agroinfiltration of *N. benthamiana*

Agroinfiltration assays, each repeated at least three times, were carried out with *A. tumefaciens* Agl1 strains, essentially as described by van Poppel *et al*. (2008). The final optical density at 600 nm (OD_600_) of the bacterial culture used for infiltration was 1.0. For co‐infiltration, bacterial cultures were mixed in a 1 : 1 ratio until a final OD_600_ of 1.0 was reached. Leaves of 4–6‐week‐old *N. benthamiana* plants were infiltrated on the lower side and the area was marked. Positive and negative controls were Agl1 strains carrying *NPP1* (Wang *et al*., 2015), the empty vector (EV) or *GUS* (Du *et al*., 2015). Infiltrations with CaCl_2_, Ca(NO_3_)_2_, MnSO_4_, MgCl_2_, ZnCl_2_ and/or LaCl_3_ were executed 24 h after agroinfiltration, covering the same area as the agroinfiltration.

Responses were monitored between 3 and 6 days after agroinfiltration. For fluorescence microscopy, a Nikon 90i epifluorescence microscope was used, equipped with a digital imaging system (Nikon DS‐5Mc camera, Nikon NIS‐AR software, Nikon Nederland, Amsterdam, The Netherlands). Autofluorescence was detected using a green fluorescent protein (GFP) filter cube (GFP‐LP, EX [Excitation] 460‐500 nm, DM [Dichroic mirror] 505 nm, BA [Barrier filter] 510 nm). Images were taken randomly from each infiltrated area. For protein isolation, agroinfiltrated *N. benthamiana *leaves were harvested at 3 days after agroinfiltration. For infection of the agroinfiltrated leaves, the leaves were detached 2 days after agroinfiltration, inoculated with *P. infestans *strain 14‐3‐GFP (20 µL of 1 × 10^4^ zoospores/mL) and incubated as described above. Lesion size was determined by measuring the horizontal and vertical diameter of each lesion, and then calculating the lesion area in mm^2^. To estimate the number of sporangia produced on each lesion, one leaf disc of 0.9 cm in diameter was submerged in 1 mL of MQ (Milli‐Q) water in a 1.5‐mL Eppendorf tube. After vortexing for about 1 min, sporangia were counted using a haemocytometer.

### Protein extraction and immunoblotting

Leaves were ground in liquid nitrogen in the presence of RIPA extraction buffer [50 mm Tris‐HCl, pH 8, 150 mm NaCl, 1% IGEPAL CA‐630 (NP‐40), 0.5% sodium deoxycholate, 0.1% sodium dodecylsulfate (SDS)]. The samples were boiled for 5 min in SDS loading buffer before being loaded on a 12% sodium dodecylsulfate‐polyacrylamide gel electrophoresis (SDS‐PAGE) gel. After electrophoresis, the proteins were transferred to Immune‐Blot polyvinylidene difluoride membranes (Bio‐Rad). For the detection of HA‐tagged proteins, the membranes were incubated for 1 h at room temperature with horseradish peroxidase (HRP)‐conjugated HA antibodies diluted 1 : 5000 in PBS‐T (Phosphate Buffered Saline with Tween 20) with 5% skimmed milk (Promega). The HA‐protein signals were detected using West Femto Chemiluminescent Substrate (Thermo Scientific, Breda, The Netherlands). Photographs were taken by UV imaging in a ChemiDoc MP system (Bio‐Rad). The proteins blotted on the membranes were stained with Coomassie brilliant blue R 250 to check whether the amount of protein loaded in each lane was in the same range.

## Supporting information


**Fig. S1** Phylogenetic tree of phospholipase D (PLD)‐likes and sPLD‐likes in *Phytophthora infestans* and characteristics of (s)PLD‐likes used in this study. Based on Meijer *et al*. (2011).

**Fig. S2** The calcium antagonist lanthanum blocks the cell death‐promoting effect of calcium. Epifluorescence photographs of *Nicotiana benthamiana* leaves at 7 days after agroinfiltration with *Agrobacterium tumefaciens* Agl1 carrying small phospholipase D (PLD)‐like constructs (PLD‐like‐1, sPLD‐like‐1 and sPLD‐like‐12) and a control construct EV (empty vector). P19 was co‐expressed and 2 mm CaCl_2_ and/or 100 μm LaCl_2_ was infiltrated 24 h after agroinfiltration. Dead cells are depicted in yellow–green and living cells in red. Experiments were repeated three times. Scale bars represent 500 μm.

**Fig. S3** Haemagglutinin (HA)‐tagged versions of small phospholipase D (PLD)‐likes induce cell death in *Nicotiana benthamiana.* (A) Epifluorescence photographs of *N. benthamiana *leaves at 7 days after agroinfiltration with *Agrobacterium tumefaciens* Agl1 carrying 3HA‐tagged versions of small PLD‐likes constructs (PLD‐like‐1, sPLD‐like‐1 and sPLD‐like‐12) and the control constructs EV (empty vector) and NPP. (B) Epifluorescence photographs of *N. benthamiana* leaves expressing 3HA‐tagged versions of PLD‐like‐1 with intact or mutated HKD motifs at 7 days post‐infiltration. P19 was co‐expressed and 2 mm CaCl_2_ and/or 100 μm LaCl_2_ was infiltrated at 24 h after agroinfiltration. Dead cells are depicted in green and living cells in red. Experiments were repeated three times. Scale bars represent 500 μm. (C) Western blot of small PLD‐likes at 3 days post‐infiltration. (D) Western blot of PLD‐like‐1 with altered HKD motifs at 3 days post‐infiltration. Proteins were detected with anti‐HA antibody.

**Fig. S4** Co‐infiltrations with CaCl_2_ and P19 do not play a role in the promotion of lesion size and sporulation of *Phytophthora infestans *on infiltrated *Nicotiana benthamiana *leaves. Growth (A) and sporulation (B) of *P. infestans *on leaves infiltrated with small phospholipase D (PLD)‐likes, calculated as the fold change in lesion size or amount of sporangia, respectively, compared with that on leaves infiltrated with the control construct (empty vector, EV). Experiments were repeated three times. Error bars represent standard deviation (*n* = 24).

**Table S1** Primers used in this study.
