# Supplementary figures and images for: Phytophthora infestans small phospholipase D‐like proteins elicit plant cell death and promote virulence
Source: Mol Plant Pathol. 2018 Oct 16;20(2):180–93. doi: 10.1111/mpp.12746 (PMC6637911; doi:10.1111/mpp.12746)

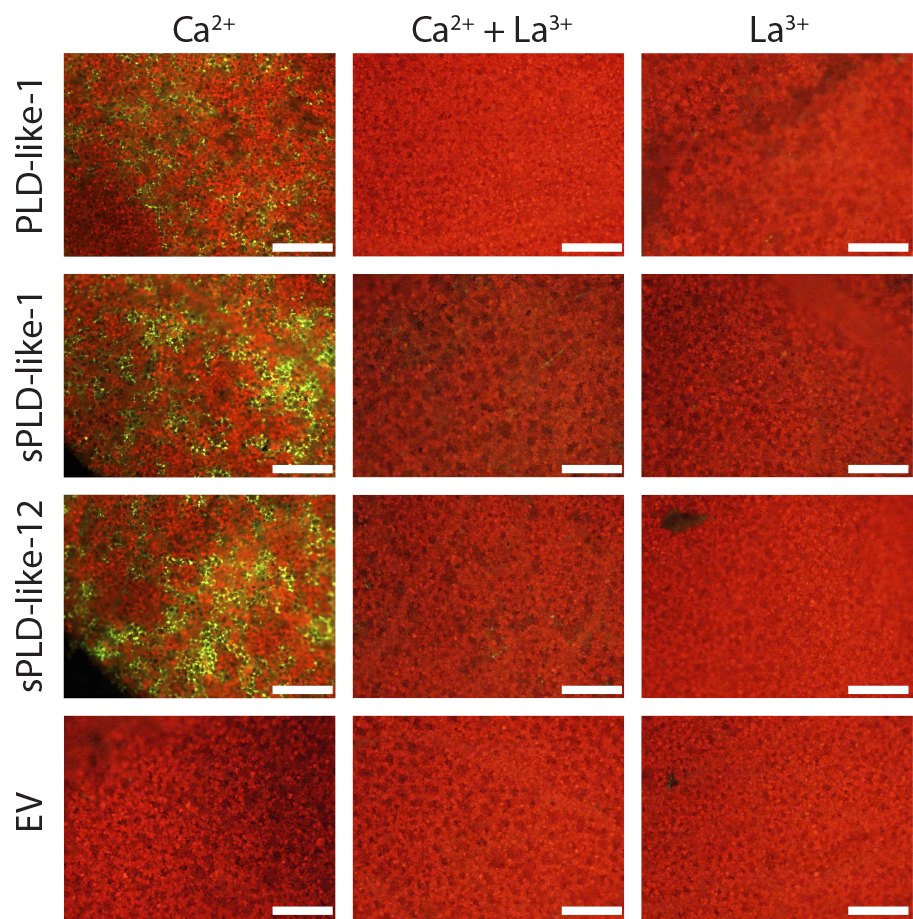

Supplement: Supplementary file 2 — Fig. S2 The calcium antagonist lanthanum blocks the cell death‐promoting effect of calcium. Epifluorescence photographs of Nicotiana benthamiana leaves at 7 days after agroinfiltration with Agrobacterium tumefaciens Agl1 carrying small phospholipase D (PLD)‐like constructs (PLD‐like‐1, sPLD‐like‐1 and sPLD‐like‐12) and a control construct EV (empty vector). P19 was co‐expressed and 2 mm CaCl2 and/or 100 μm LaCl2 was infiltrated 24 h after agroinfiltration. Dead cells are depicted in yellow–green and living cells in red. Experiments were repeated three times. Scale bars represent 500 μm. [file MPP-20-180-s002.tif]

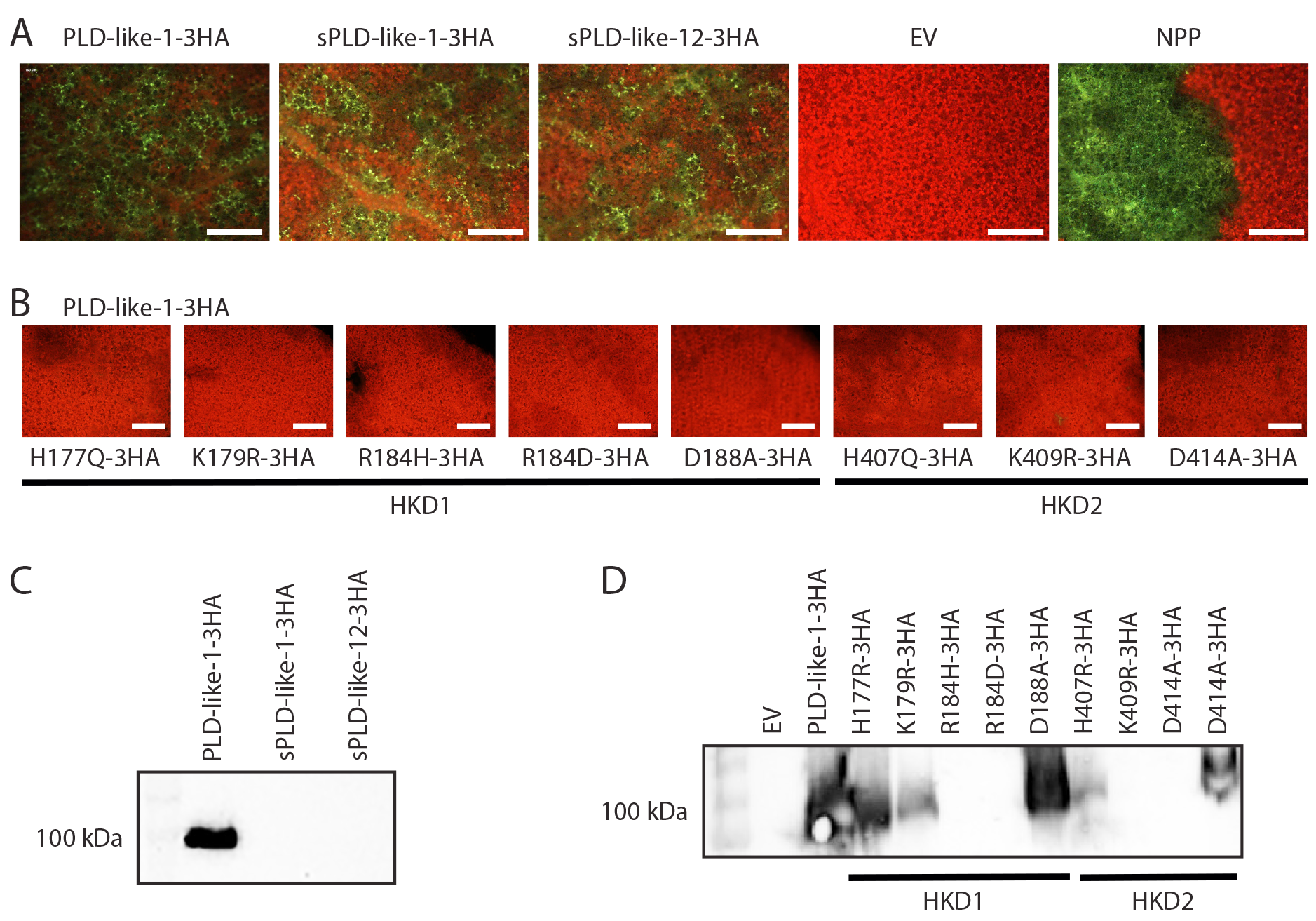

Supplement: Supplementary file 3 — Fig. S3 Haemagglutinin (HA)‐tagged versions of small phospholipase D (PLD)‐likes induce cell death in Nicotiana benthamiana. (A) Epifluorescence photographs of N. benthamiana leaves at 7 days after agroinfiltration with Agrobacterium tumefaciens Agl1 carrying 3HA‐tagged versions of small PLD‐likes constructs (PLD‐like‐1, sPLD‐like‐1 and sPLD‐like‐12) and the control constructs EV (empty vector) and NPP. (B) Epifluorescence photographs of N. benthamiana leaves expressing 3HA‐tagged versions of PLD‐like‐1 with intact or mutated HKD motifs at 7 days post‐infiltration. P19 was co‐expressed and 2 mm CaCl2 and/or 100 μm LaCl2 was infiltrated at 24 h after agroinfiltration. Dead cells are depicted in green and living cells in red. Experiments were repeated three times. Scale bars represent 500 μm. (C) Western blot of small PLD‐likes at 3 days post‐infiltration. (D) Western blot of PLD‐like‐1 with altered HKD motifs at 3 days post‐infiltration. Proteins were detected with anti‐HA antibody. [file MPP-20-180-s003.tif]

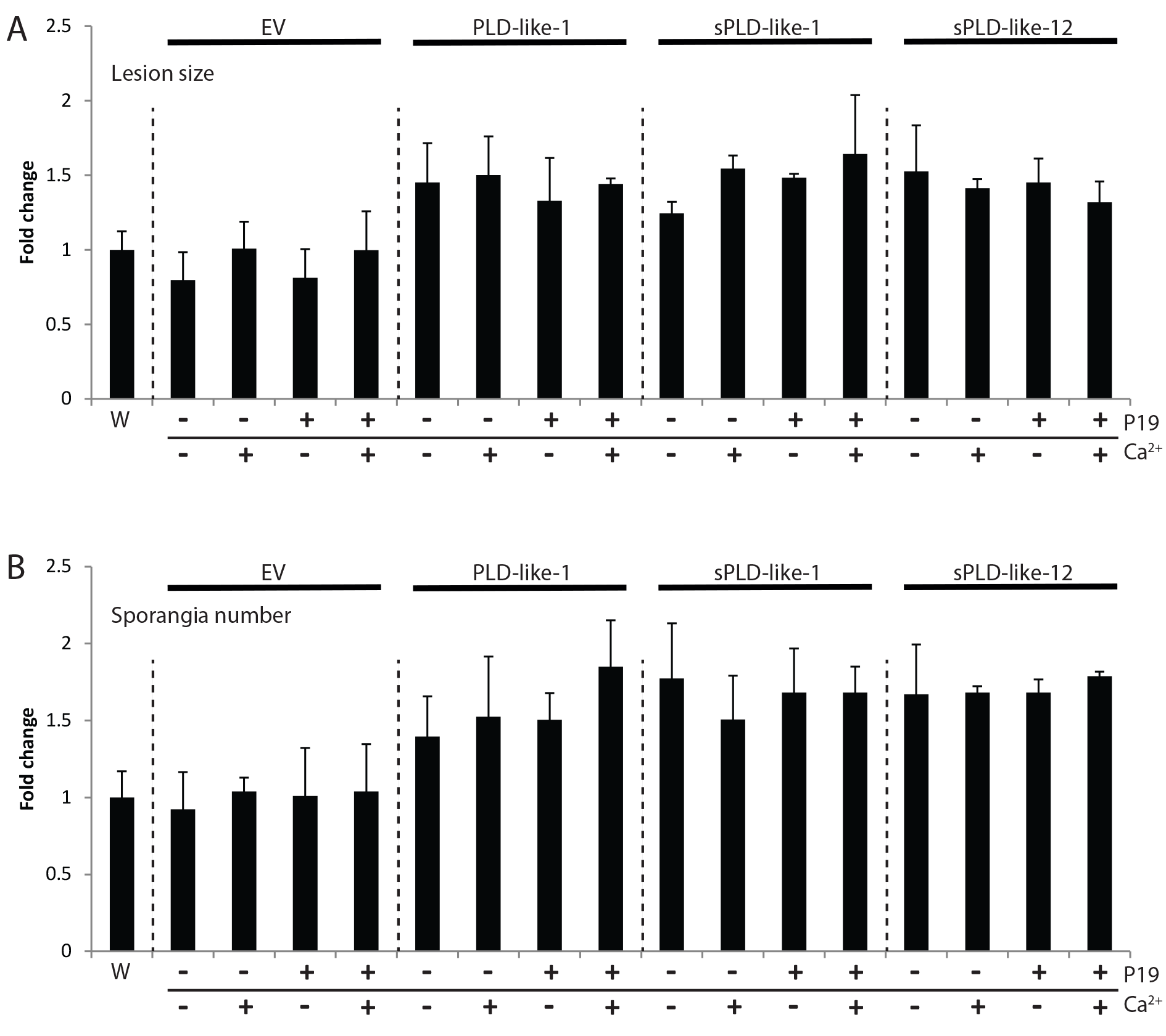

Supplement: Supplementary file 4 — Fig. S4 Co‐infiltrations with CaCl2 and P19 do not play a role in the promotion of lesion size and sporulation of Phytophthora infestans on infiltrated Nicotiana benthamiana leaves. Growth (A) and sporulation (B) of P. infestans on leaves infiltrated with small phospholipase D (PLD)‐likes, calculated as the fold change in lesion size or amount of sporangia, respectively, compared with that on leaves infiltrated with the control construct (empty vector, EV). Experiments were repeated three times. Error bars represent standard deviation (n = 24). [file MPP-20-180-s004.tif]
