# Supplementary material for: Phytophthora infestans small phospholipase D‐like proteins elicit plant cell death and promote virulence
Source: Mol Plant Pathol. 2018 Oct 16;20(2):180–93. doi: 10.1111/mpp.12746 (PMC6637911; doi:10.1111/mpp.12746)
Supplement: Supplementary file 5 — Table S1 Primers used in this study. [file MPP-20-180-s005.docx]

**Table S1.** Primers used in this study.

| **Cloning** | | | | |
| --- | --- | --- | --- | --- |
| **Gene** | **Forward primer** | **Sequence (5’-3’)** | **Reverse primer** | **Sequence (5’-3’)** |
| *PLD-like-1* | PLDL1-H177Q-F | CGTCGCACCAGCAGAAGTCG | PLDL1-H177Q-R | CGACTTCTGCTGGTGCGACG |
|  | PLDL1-K179R-F | CACCACCAGAGGTCGGTGATC | PLDL1-K179R-R | GATCACCGACCTCTGGTGGTG |
|  | PLD1-R184D-F | GTGATCGTGGATAAGGGCCGTG | PLD1-R184D-R | CACGGCCCTTATCCACGATCACC |
|  | PLDL1-D188A-F | GGGCCGTGCTCTCGTCGC | PLDL1-D188A-R | CGAGAGCACGGCCCTTACG |
|  | PLDL1-H407Q-F | GTACATCCAGAGTAAGCTGG | PLDL1-H407Q-R | GCTTACTCTGGATGTACAGC |
|  | PLDL1-K409R-F | CACAGTAGGCTGGTGATTG | PLDL1-K409R-R | CACCAGCCTACTGTGGATG |
|  | PLDL1-D414A-F | GATTGTCGCCGACGTGTAC | PLDL1-D414A-R | CACGTCGGCGACAATCAC |
|  | PLD1-F-HA | CGTTGAGGGGGATGACGGGTTAATTAACATC | PLD1-R-HA | GATGTTAATTAACCCGTCATCCCCCTCAACG |
| *sPLD-like-1* | sPLD1-H208Q-F | GCGGTATTTTACAGCAGAAGATATGG | sPLD1-H208Q-R | CCATATCTTCTGCTGTAAAATACCGC |
|  | sPLD1-K210R-F | GTATTTTACATCAGAGGATATGGATCTTTGAC | sPLD1-K210R-R | GTCAAAGATCCATATCCTCTGATGTAAAATAC |
|  | sPLD1-D215V-F | Gatatggatctttgtcgacagtcac | sPLD1-D215V-R | GTGACTGTCGACAAAGATCCATATC |
|  | sPLD1-H484Q-F | GCGTGAATCAGGCCAAGTACATTG | sPLD1-H484Q-R | CAATGTACTTGGCCTGATTCACGC |
|  | sPLD1-K486R-F | GTGAATCATGCCAGGTACATTGTCAC | sPLD1-K486R-R | GTGACAATGTACCTGGCATGATTCAC |
|  | sPLD1-D491A-F | CATTGTCACCGTCACTCGCGTG | sPLD1-D491A-R | CACGCGAGTGACGGTGACAATG |
|  | PR1-sPLD1-F | GCAATTTCACGAGGAGTGAAGCGGGCACGGCAAGAGTGGGATATTAC | sPLDL1-R | gccGcggccgcttacgattcagcagatacaat |
|  | sPLDl-mSP-F | cggaattcATGcgcttcactcctcgtgaa |  |  |
|  | sPLD1-F-HA | GAAATTGTATCTGCTGAATCGGGGTTAATTAACATC | sPLD1-R-HA | GATGTTAATTAACCCCGATTCAGCAGATACAATTTC |
| *sPLD-like-12* | sPLDL12-H246Q-F | CAACTCACCAGCAGAAGAATC | sPLDL12-H246Q-R | GATTCTTCTGCTGGTGAGTTG |
|  | sPLDL12-K248R-F | CCAGAGGAATCTAATCATAGC | sPLDL12-K248R-R | GCTATGATTAGATTCCTCTGG |
|  | sPLD-12-A253D-F | CTAATCATAGACTCTACCAATTCGACG | sPLD-12-A253D-R | CGTCGAATTGGTAGAGTCTATGATTAG |
|  | sPLDL12-D261A-F | GGTGGCTCAACACCCTG | sPLDL12-D261A-R | GTGTTGAGCCACCTCCG |
|  | sPLDL12-H483Q-F | CATCCAGTCAAAAGTTGTGATC | sPLDL12-H483Q-R | CAACTTTTGACTGGATGTACAG |
|  | sPLDL12-K485R-F | CACTCAAGAGTTGTGATCATC | sPLDL12-K485R-R | CACAACTCTTGAGTGGATG |
|  | sPLDL12-D490R-F | GATCATCGCCGACGTGTAC | sPLDL12-D490R-R | CACGTCGGCGATGATCAC |
|  | PR1-sPLD12-F | CGACTGACTGTGGAGAGAATTGAGGGCACGGCAAGAGTGGGATATTAC | sPLD12-R | gccGcggccgcctacgtgcaggtgtcctgtg |
|  | sPLD12-mSP-F | gccactagtatggttcgccatctagg |  |  |
|  | sPLD12-F-HA | CCACAGGACACCTGCACGGGGTTAATTAACATC | sPLD12-R-HA | GATGTTAATTAACCCCGTGCAGGTGTCCTGTGG |
| **Expression analysis** | | | | |
| **Gene** | **Forward primer** | **Sequence (5’-3’)** | **Reverse primer** | **Sequence (5’-3’)** |
| *ActA* | ActA-F | CATCAAGGAGAAGCTGACGTACA | ActA-R | GACGACTCGGCGGCAG |
| *PLD-like-1* | qPLD1-F | CCAGAAGTTCATGGAGGCGAC | qPLD1-R | GCCGTCGTCGTGCGCAGC |
| *sPLD-like-1* | qsPLD1-F | TGGGGATATTTCTACACCACGG | qsPLD1-R | GCAGATACAATTTCCTCCAAGCTT |
| *sPLD-like-12* | qsPLD12-F | ctgccaacatcattgacga | qsPLD12-R | taaacggcatagcgtccaa |
